# The "Red Chamber" Treatment of Small-Pox Patients

**Published:** 1894-11-03

**Authors:** 


### EDITOR'S LETTER-BOX.

[Our correspondents are reminded that prolixity is a great bar to publication, and that brevity of style and conciseness of statement greatly facilitate early insertion.]

### THE "RED CHAMBER" TREATMENT OF SMALL-POX PATIENTS.

"A Correspondent" writes: Last winter some experiments were made at the Oresund Hospital in Copenhagen in treating small-pox patients in a "red chamber," that is, a room where daylight is kept out so as to prevent the chemical rays (the blue-violet rays) from acting upon the skin. This was done at the instance of a young Danish doctor, who had previously ascertained that the chemical rays of the sun could produce inflammation of healthy skin, and he naturally concluded that the skin of small-pox patients might probably be still more susceptible, as these patients generally get the deepest and largest marks on those parts of the body which are most exposed to daylight, viz., the face and the hands. In the beginning of 1894 there was a slight small-pox epidemic in Copenhagen, which afforded sufficient opportunities for testing practically the theory of the "red chamber," and there cannot be the slightest doubt but that it will prove of the greatest service in the treatment of this disease. The method was applied at the Oresund and the West Hospital, Copenhagen, in the following manner: In order to deprive the daylight of all its blue-violet rays, there were placed three layers of red shirting and one of yellow before every window; the gas lights were fitted with red glasses, same as used by the photographers, and there were put red curtains over the windows to the corridors and red portières over the doors. Red window panes were subsequently substituted in order to make the rooms lighter, but it was found necessary to have red cotton in front. At the meals and during the doctor's visits stearine candles were used. The result was very satisfactory, the patients who were received during the earlier stages of their illness not only got very quickly through, avoiding the worst days of the illness and having but little fever (four to five days), but they also escaped from being inflicted with the well-known marks. The treatment should be commenced as early as possible, before the pustules have shown themselves, whereby the latter grow more slowly and do not become so large. Altogether fourteen patients were treated in Copenhagen, and although this is only a small number, the results fully confirmed what has been said above.

